# An Ongoing Role for Structural Sarcomeric Components in Maintaining *Drosophila melanogaster* Muscle Function and Structure

**DOI:** 10.1371/journal.pone.0099362

**Published:** 2014-06-10

**Authors:** Alexander D. Perkins, Guy Tanentzapf

**Affiliations:** Department of Cellular and Physiological Sciences, University of British Columbia, Life Sciences Institute, Vancouver, British Columbia, Canada; Technische Universität Dresden, Germany

## Abstract

Animal muscles must maintain their function while bearing substantial mechanical loads. How muscles withstand persistent mechanical strain is presently not well understood. The basic unit of muscle is the sarcomere, which is primarily composed of cytoskeletal proteins. We hypothesized that cytoskeletal protein turnover is required to maintain muscle function. Using the flight muscles of *Drosophila melanogaster*, we confirmed that the sarcomeric cytoskeleton undergoes turnover throughout adult life. To uncover which cytoskeletal components are required to maintain adult muscle function, we performed an RNAi-mediated knockdown screen targeting the entire fly cytoskeleton and associated proteins. Gene knockdown was restricted to adult flies and muscle function was analyzed with behavioural assays. Here we analyze the results of that screen and characterize the specific muscle maintenance role for several hits. The screen identified 46 genes required for muscle maintenance: 40 of which had no previously known role in this process. Bioinformatic analysis highlighted the structural sarcomeric proteins as a candidate group for further analysis. Detailed confocal and electron microscopic analysis showed that while muscle architecture was maintained after candidate gene knockdown, sarcomere length was disrupted. Specifically, we found that ongoing synthesis and turnover of the key sarcomere structural components Projectin, Myosin and Actin are required to maintain correct sarcomere length and thin filament length. Our results provide *in vivo* evidence of adult muscle protein turnover and uncover specific functional defects associated with reduced expression of a subset of cytoskeletal proteins in the adult animal.

## Introduction

In order to accomplish the diverse functions required for life, the tissues of multicellular organisms must be arranged into complex, three-dimensional structures. Once formed this tissue organization must be maintained throughout the life of the organism. Understanding how these two processes, development and maintenance, are accomplished is a key aim of cell and developmental biology. One such tissue is muscle, which must withstand continuous mechanical and chemical stresses while maintaining function. Muscles provide an excellent model in which to study tissue maintenance because they persist throughout the life of the organism and are organized into a highly structured, near crystalline, architecture [1], [2]. For example, vertebrate cardiac muscle cells live for many decades [3] and fly muscle cells survive for the entire lifespan of the adult animal [4].

The fruit-fly, *Drosophila melanogaster*, provides an excellent model system in which to study muscle tissue maintenance: there is no adult myogenesis in the fly, flies are amenable to genetic analysis, mutations in many sarcomeric components are available, fly sarcomeric proteins are well-conserved in relation to their vertebrate orthologs, and flies possess a number of well-characterized muscle types. Myogenesis in the fly is restricted to two phases of development: embryonic myogenesis, in which larval muscles are formed from precursors; and pupal myogenesis, in which nearly all larval muscles are destroyed and adult muscles are formed [5]. After pupal myogenesis no new muscles are made, so adult muscles must be maintained for the whole *D. melanogaster* lifespan [4]. Genetic screens have identified loss-of-function mutations in the genes encoding all the major sarcomeric components [6]–[8]. The core sarcomeric components of fly muscles are, for the most part, well-conserved in comparison to vertebrates [9]–[12]. A number of different muscle types in flies have been used to study the function of the cytoskeletal and sarcomeric components: embryonic muscles during the first round of fly myogenesis [5], [13]–[15], the leg muscles during the second round of fly myogenesis [16], [17], and the heart muscles as a model for cardiac function [18]. One of the best-studied muscles in the adult fly is the indirect flight muscle (IFM), which powers flight [6], [19], [20]. Since the IFMs are not required for viability and their function is easily assayed, they have become an important model system for the identification and characterization of essential muscle genes. Thus the adult fly musculature, and the IFMs in particular, provides a powerful model system for studying muscle maintenance since it is post-mitotic; easily accessible for mechanical, physiological and behavioral assays; and, amenable to many different genetic techniques [20].

Understanding how muscles are maintained throughout the lifetime of an organism has direct implications on our understanding of myodegenerative diseases and aging. Work in both animal models and clinical studies of human patients have identified a number of genes that are required for muscle maintenance. This work has highlighted two broad categories of genes that are involved in muscle maintenance: cytoskeletal and sarcomeric genes, and oxidative stress-related genes. Animal models have been useful in studying the roles of cytoskeletal components in maintaining muscle structure and function [4], [8]. Studies using hypomorphic alleles of the sarcomeric proteins Myosin Heavy Chain (Mhc) [21], Flightin [22], [23] and Troponin T [24]; mutational analysis of the costameric components Sarcoglycan [25], Dystroglycan and Dystrophin [26], and integrin [27] have all shown that these genes play essential roles in maintaining muscle function. Genetic analysis of human patients also identified a number of cytoskeletal and sarcomeric genes as being required for adult muscle function; mutations in actin, Troponin, Tropomyosin, Myosin and Nebulin have been implicated in congenital myopathies [28]. Moreover, mutations in the proteins Myotilin and Titin cause limb-girdle muscular dystrophy 1A and tibial muscular dystrophy respectively [29], [30]. The second group, the oxidative stress-related genes, typically causes disruption to the equilibrium between muscle damage and muscle repair leading to an accumulation of damage in muscle tissue. Such mutations impinge on oxidative stress homeostasis rather than the disruption of core contractile machinery [31]. Excess oxidative stress in the mitochondria of adult *D. melanogaster* muscles has been shown to lead to myodegeneration [32], [33]. Furthermore, disruptions to pathways that limit oxidative damage in mice exacerbate the effects of muscular dystrophy [34]. In humans, increased oxidative stress due to Vitamin E deficiencies [35] or defective antioxidase enzymes [36] are also linked with increased myodegeneration and muscular dystrophy [31].

Although mutations that affect muscle function in the adult fly have been previously identified, it is presently unclear whether these phenotypes are due to defects in muscle maintenance. In many cases it is likely that the defects occured during myogenesis and are only revealed during adulthood [26], [37]–[40]. Thus, the main problem in studying how adult muscle structure and function is maintained lies in describing functions in fully formed muscles for genes whose activity was required to form the muscles [21]–[24], [41]. Importantly, this issue has prevented the execution of a systematic screen to identify genes that are specifically required for the long-term maintenance of muscle function in the adult. This is despite the fact that a comprehensive RNAi screen was used to define the complete set of genes required for *D. melanogaster* muscle function, as this screen used an approach which knocked-down genes throughout development [41]. We circumvented this problem by using temporal and tissue specific expression of RNAi constructs to limit gene knockdown to adult fly muscle and then assaying their climbing ability using a negative geotaxis assay. With this approach we were able to systematically analyze the role of the cytoskeleton in *D. melanogaster* muscle maintenance [42]. This screen identified 46 genes required to maintain muscle function in adult flies. Of these 46 genes, 40 had no previously known role in adult muscle maintenance. Here we demonstrate that a subset of cytoskeletal proteins, specifically key components of the sarcomere, are continuously expressed and undergo turnover in muscle through the life of the fly. Furthermore, failure to renew these cytoskeletal components results in both functional and morphological muscle defects.

## Results

### Adult fly muscles exhibit ongoing transcription of sarcomeric proteins with morphological stability

**Figure 1:**
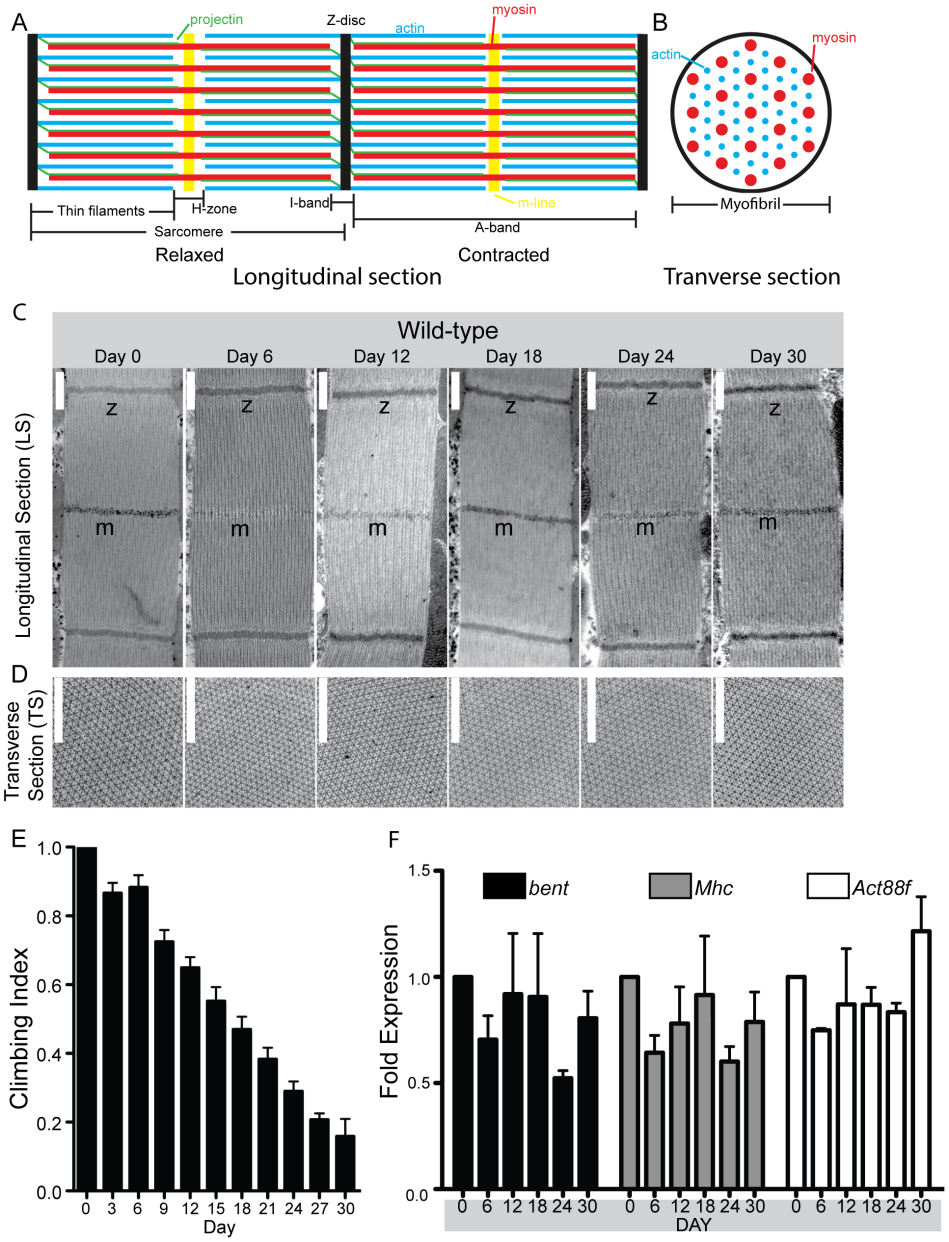
Characterization of adult muscle. The morphology, function and transcriptional activity of wild-type indirect flight muscles (IFM) was assayed at sequential time points during the first 30 days of fly life. (A) Simplified diagram showing the relaxed and contracted state of sarcomeres in a longitudinal section. The structural elements relevant to the work presented in this study have been included. Actin, or thin, filaments are shown in blue; Myosin, or thick, filaments are shown in red; Projectin is shown in green; the M-line is shown in yellow; and the Z-disc is shown in black. Note the slight shortening of the sarcomere in the contracted state, specifically in the H-zone and I-band as is characteristic of the IFM. (B) Simplified diagram of a transverse section of a sarcomere. As in (A), actin filaments are shown in blue and myosin filaments are shown in red. (C) Longitudinal Sections (LS) and (D) Transverse Sections of wild-type IFM sarcomeres show that the Z-disc, M-lines, actin and myosin filaments, and the myosin and actin lattice all exhibit consistent appearance over the first 30 days of fly life. (C: z, Z-disc. m, M-line; D: Larger circles are myosin, smaller dots are actin; Scale bars are in white and are 500 nm; see Figure 1A for schematic representations). (E) The climbing ability of flies was assayed using a negative geotaxis assay. Over the 30 day time course, the climbing ability of adult flies decreased in a linear fashion by 82%. N is 10 independent replicates each using 10 flies to assay climbing ability. (F) Key sarcomeric genes are continually transcribed in adult flies. RNA was extracted from whole flies and qPCR was used to assay for expression levels of *bent*, *Mhc*, and *Act88F*. GAPDH was used as the internal control for expression. Expression levels were normalized to the Day 0 timepoint. For all three genes, mRNA levels remained close, with slight variations, to the initial timepoint over the first 30 days of fly life. N≥3 for all qPCR time points, where each replicate was an independent RNA extraction using 10 flies.

During aging, fly muscles undergo characteristic changes including: Z-disc disruption/fragmentation, reduction in the expression of sarcomeric components, loss of thin filament organization, and reduced flight and climbing ability. However, these studies focused on flies towards the end of their lifespan [43]. To test whether muscles also undergo changes earlier in life, we investigated muscle morphology, function, and sarcomeric protein expression prior to the time at which aging-associated defects appear. Electron microscopy was used to analyze the ultrastructure of the indirect flight muscle (IFM) at 6-day intervals following eclosion. Longitudinal and transverse sections revealed no obvious changes in sarcomeric architecture between Day 0 and Day 30 (Figure 1A-D). Next, we assayed muscle function over the first 30 days post eclosion using a classic behavioral assay to test climbing ability. We observed a functional decline starting soon after eclosion which resulted in an 82% decline in climbing ability over 30 days (Figure 1E). We then tested, using quantitative PCR (qPCR), whether the transcription of core sarcomeric proteins varied during the fly lifespan. We assayed mRNA levels of *bent* (Projectin), *Mhc* (muscle Myosin), *Act88F* (Actin) at 6-day intervals for 30 days. All three core sarcomeric proteins were continuously transcribed from eclosion until Day 30 (Figure 1F). Taken together our data reveal that during the first 30 days of fly life muscles are a transcriptionally and functionally dynamic, but structurally static, tissue.

### Actin undergoes *in vivo* turnover in adult muscles

**Figure 2:**
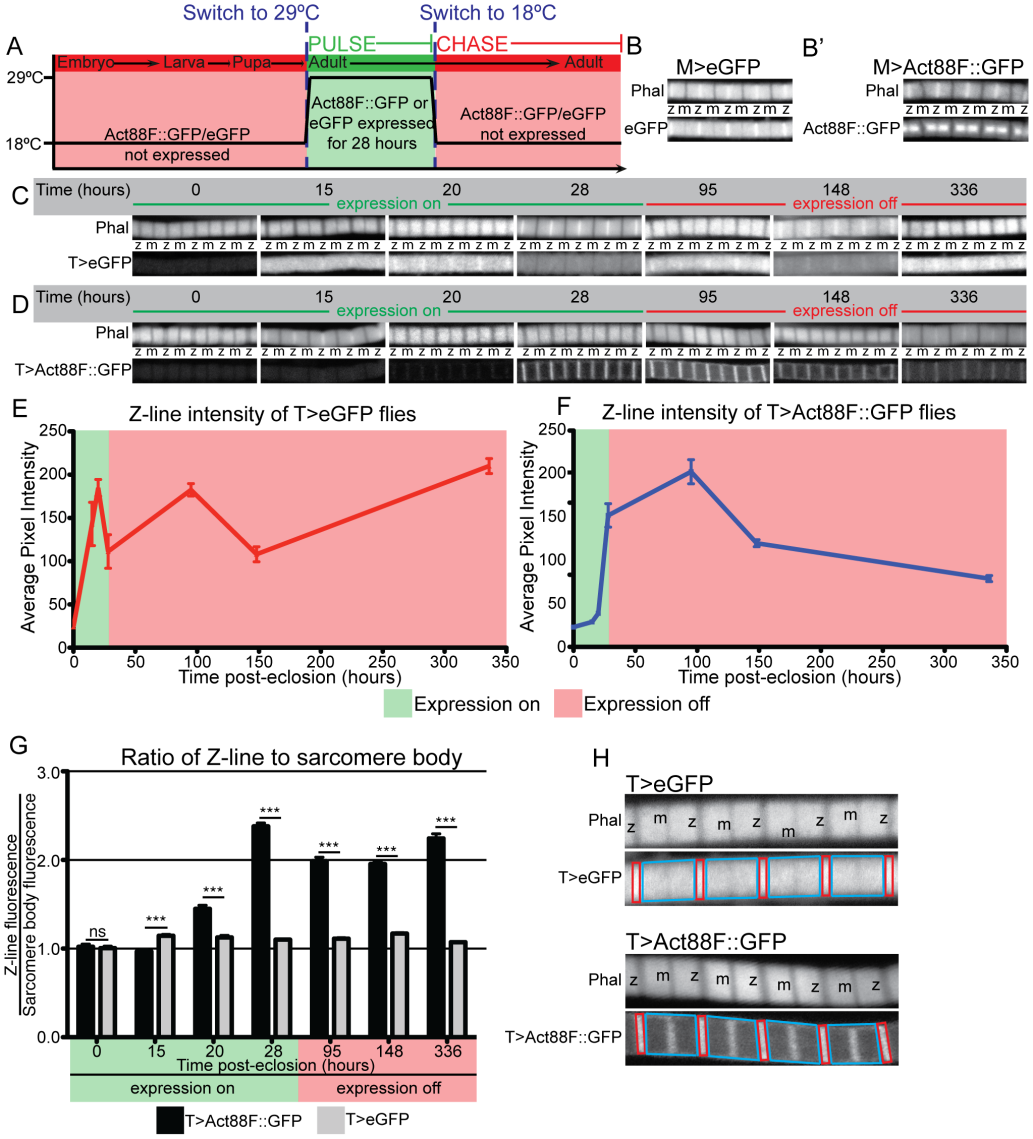
Sarcomeric actin undergoes turnover in adult muscles. (A) Graphic representation of the experimental design for the pulse-chase experiments. Act88F::GFP or eGFP was expressed under the control of the TARGET system for 28 hours directly after eclosion (the pulse) and then shut off (the chase). (B, B′) Localization of eGFP (B) or Act88F::GFP (B′) when expressed throughout development and adulthood with the Mef2:GAL4 system (F-actin is counterstained with rhodamine-phalloidin). eGFP non-specifically labels the whole sarcomere while Act88F::GFP is specifically localized to the sarcomeric core. (C,D) A time series of representative images of IFMs from flies expressing eGFP (C) or Act88F::GFP (D) under the control of the TARGET system (F-actin counterstained with rhodamine-phalloidin). The locations of the Z-discs and M-lines are indicated by a ‘z’ or ‘m’ in both panels. (E) Quantification of fluorescent intensity at the Z-disc for eGFP during TARGET pulse-chase experiment. Intensity at the Z-disc increased during the 28 hr pulse phase but did not decline afterwards. Instead, intensity remained elevated. (F) Quantification of fluorescent intensity at the Z-disc for Act88F::GFP during TARGET pulse-chase experiment. Intensity at the Z-disc increased during the 28 hour pulse phase and then gradually declined. (G) The ratio of Z-disc to sarcomere body fluorescent intensity during the pulse-chase experiment shows that the increase in staining intensity is specific to the Z-disc for Act88F::GFP. Comparison of Z-disc to sarcomere body intensity ratios for eGFP (grey bars) and Act88F::GFP (black bars) expression shows that while eGFP localization is non-specific, Act88F::GFP is significantly enriched at the Z-disc. (H) Examples showing how Z-disc and sarcomere intensity was measured for both TARGET>eGFP and TARGET>Act88F::GFP myofibrils. Phalloidin counterstains were used to identify relevant areas. Red rectangles indicate the area quantified for Z-disc intensities. Irregular blue tetragons indicate the area quantified for sarcomere body intensities. For (E,F,G) N≥40 individual sarcomeres from ≥5 animals. For all panels, error bars indicate standard error; ns indicates not significant; * indicates a p-value<0.05, ** indicates a p-value<0.005, *** indicates a p-value<0.0005. TARGET>eGFP and TARGET>Act88F::GFP are abbreviated as T>eGFP and T>Act88F::GFP throughout.

As our qPCR analysis showed persistent expression of sarcomeric actin during fly life, we asked whether sarcomeres undergo renewal through protein turnover. A pulse-chase approach was used to study the turnover of the fly Actin88F protein; actin is the most abundant protein in muscle fibres [44] and the *Act88F* gene was selected for this experiment because it is specifically restricted to the sarcomeres in IFMs [45]. To generate pulsed expression of a GFP-tagged Act88F (Act88F::GFP), or cytoplasmic eGFP control (eGFP), the TARGET system was used; TARGET (Temporal And Regional Gene Expression Targeting [46]) allows the induction of tissue specific expression in muscle through a simple temperature shift (from 18°C to 29°C) (Figure 2A). Optimal labeling was obtained with a 28 hr pulse of expression starting directly post-eclosion. This was sufficient to produce equivalent maxima of intensity to those observed when the pulse of expression was not followed by a chase but was not so long as to cause fluorescent saturation (see Materials and Methods). The localization of Act88F::GFP when expressed only in adults using the TARGET system was distinct to its localization when expressed throughout development by the Mef2:GAL4 system (compare Figure 2B′, D). The expression pattern of Act88F:GFP under control of the Mef2::GAL4 system was consistent with previous reports [47]. The localization of eGFP was similar under both conditions (compare Figure 2B-C), which is consistent with non-specific incorporation of the eGFP.

By quantifying the fluorescent intensity at the Z-disc, we showed that Act88F::GFP was incorporated into the muscles beginning 20 hours after its expression was induced (Figure 2D, F, H). After the expression of Act88F::GFP was repressed by shifting to the non-permissive temperature (at 28 hours), continued to increase until the 95 hour time point (Figure 2D,F). After 95 hours, intensity gradually decreased. At these later time points additional staining of Act88F::GFP was observed in the M-line and the muscle body, though this staining was weaker than that observed in the Z-disc (Figure 2D). The continued presence of fluorescence in the sarcomere, albeit at a declining level, after Act88F::GFP expression was repressed suggests that, once incorporated, sarcomeric proteins have a half-life of several days. In comparison, when a control pulse-chase experiment using a cytoplasmic eGFP showed that fluorescence became visible 15 hours after induction and that it remained at an elevated, fluctuating, level long after expression was repressed (Figure 2C, E).

We next demonstrated that that the incorporation of Act88F::GFP was mostly restricted to the Z-disc using a quantitative image analysis approach. We calculated the ratio of fluorescent intensity at the Z-disc compared to the fluorescent intensity of the adjacent sarcomere body (see Materials and Methods, Figure 2H). The ratio of Z-disc to sarcomere body intensity during expression of Act88F::GFP rose sharply, indicating that the Z-disc was the initial, and main, site for Act88F::GFP incorporation into the sarcomere (Figure 2D, G). Once expression of the Act88F::GFP was repressed (the chase phase) the ratio decreased slightly although the majority of the Act88F::GFP continued to localize to the Z-disc (Figure 2D, G). Comparatively, the ratio of Z-disc to sarcomere body intensity for eGFP expression showed a significantly lower Z-disc localization at all time-points (Figure 2C, G) although some Z-disc localization was evident (Figure 2C). This data argues that the Z-disc is likely the major site of initial incorporation of newly synthesized Act88F::GFP in the adult muscle and that subsequently Act88F::GFP spreads into the rest of the sarcomere. Overall the pulse-chase experiments provide direct evidence that sarcomeric actin undergoes turnover in adult fly muscles and that actin is initially incorporated into the muscle at the Z-disc.

### A screen identifying genes required to maintain muscle function

Our data thus far suggests that adult fly muscles are undergoing protein turnover at the sarcomere. Consequently, blocking the synthesis of specific proteins may have an adverse effect on muscle maintenance. In particular, we hypothesized that turnover of cytoskeletal proteins is important for muscle maintenance as the cytoskeleton forms the contractile machinery [10]. We tested our hypothesis directly by taking advantage of technical advances that make it possible to carry out a muscle specific gene knockdown screen in the adult muscles [42]. This screen was carried out in two steps: the first used the Mef2:GAL4 driver to express RNAi constructs throughout development; the second used the TARGET system, as previously discussed, to limit RNAi construct expression to adult flies [42].

An important concern when working with RNAi is the potential for off-target effects. Off-target effects could lead to false-positives in the results of the screen. To address this potentiality we tested secondary lines for 30 genes in the Mef2:GAL4 screen and 17 genes in the TARGET screen [42]. In the Mef2:GAL4 screen 93%, 28 out of 30, of the secondary lines tested confirmed the phenotype observed with the first line tested. In the TARGET screen 88%, 15 out of 17, lines confirmed the phenotype of the first line. For both screens, this represents a high level of hit confirmation, indicating that off-target effects were not a major concern for our screen. Complete details of the screen including the design rationale, raw data, analysis technique, technical validation and results can be found in our associated dataset publication [42].

### Identification of cytoskeletal genes required for muscle function

**Figure 3:**
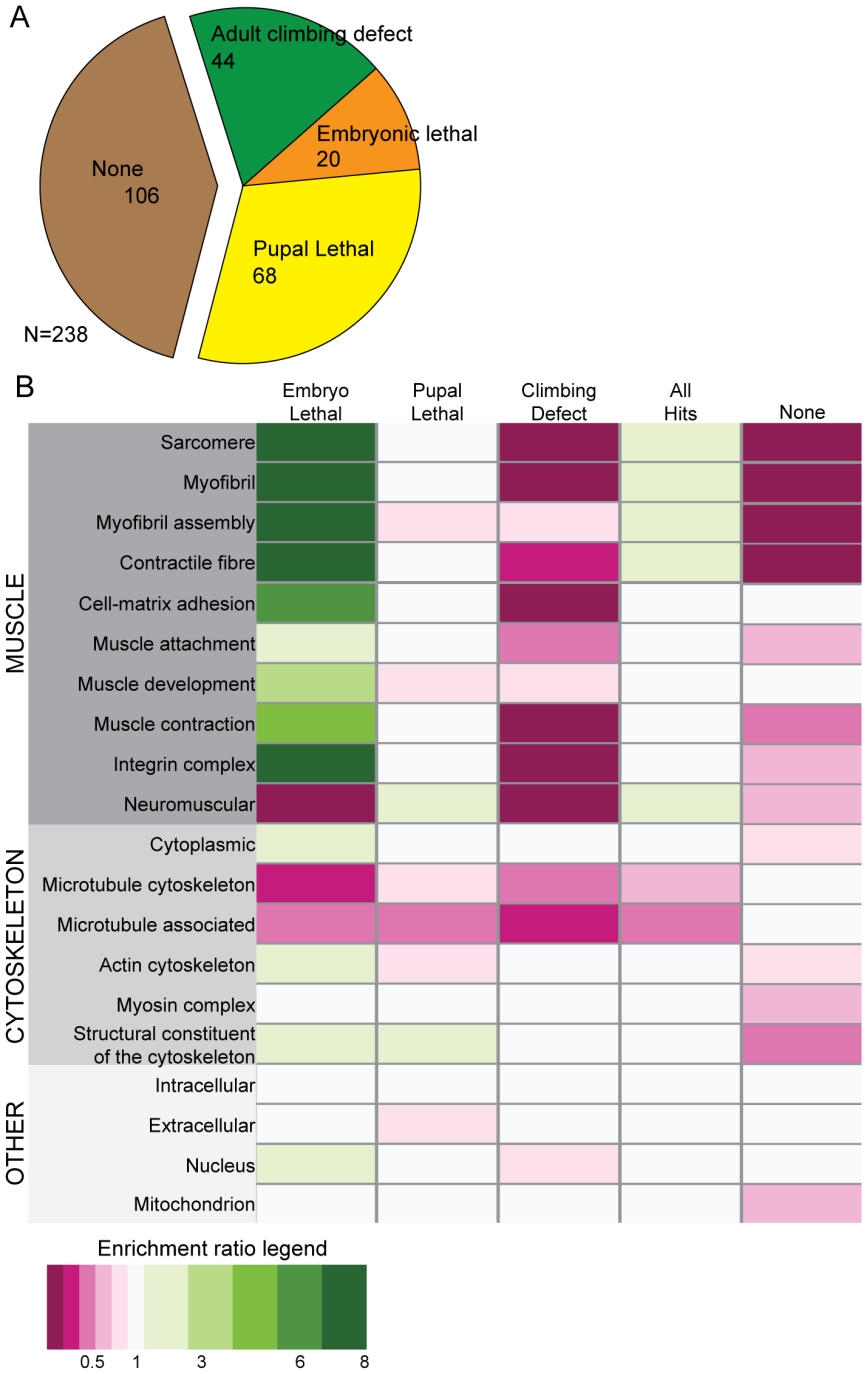
Results of the Mef2:GAL4 screen of the role cytoskeleton muscle function. (A) Phenotypic breakdown of results from the Mef2:GAL4 screen. The Mef2:GAL4 screen expressed the RNAi constructs throughout both development and adulthood. By assaying or developmental lethality or climbing ability, we observed that 106 genes had no role in muscle development or maintenance (None, brown), 20 were Embryo Lethal, 68 were Pupal Lethal, and 44 caused a Climbing Defect. (B) Enrichment analysis of Gene Ontology (GO) terms by phenotype. Enrichment ratios were calculated by comparing frequency of a term in a specific phenotypic class to the frequency of the same term in the entire screened set. GO terms are listed on the left. An enrichment ratio of 0 indicates that a given term did not appear in the phenotypic group. An enrichment ratio of <1 indicates that the frequency for the term was reduced in the phenotypic class compared to the whole screened set. An enrichment ratio of 1 indicates that the frequency for the term was the same in the phenotypic class compared to the whole screened set. An enrichment ratio of >1 indicates that the frequency for the term was the enriched in the phenotypic class compared to the whole screened set.

The Mef2:GAL4 driver was used to express 270 RNAi construct lines targeting the 238 candidate genes. Of the 238 genes tested, 150 reached adult stage (Figure 3A). The 88 genes whose knockdown resulted in lethal phenotypes were classified as either ‘Embryo lethal’ (20 genes) or ‘Pupal lethal’ (68 genes); none showed larval lethality (Figure 3A). The climbing ability of the 150 genes whose RNAi-mediated knockdown resulted in viable flies was assayed and the lines were classified as ‘Climbing defect’ (44 genes) or ‘None’ (106 genes lines) (Figure 3A). In total, the Mef2:GAL4 driven RNAi-mediated, knockdown of 132 genes caused a phenotype, indicating that they are all required for muscle function.

The distribution of Gene Ontology (GO) terms (www.geneontology.org) was analyzed by phenotypic class. An enrichment ratio was calculated that compared the prevalence of a certain GO term in a phenotypic class (e.g.: ‘Embryo lethal’) to its prevalence in the entire screened library of genes (see Materials and Methods; Figure 3B). In general, genes with cytoskeletal associated GO terms were enriched in the earlier, possibly more severe, phenotypic classes (Figure 3B). However, there were some exceptions. For example, hits in genes with microtubule-associated GO terms were rare (Figure 3B). As muscle and cytoskeletal associated terms were likely to give earlier defects they were largely absent from the ‘Climbing defect’ class, underscoring the need to use the TARGET system to analyze the function of such genes in later stages. Interestingly, the GO term ‘Neuromuscular’, while depleted in all other sets, was enriched in the ‘Pupal Lethal’ set (Figure 3B). In general, within the set of genes whose knockdown resulted in a phenotype, ‘All Hits’, the only GO terms that showed enrichment were those associated with muscle structure and development. These ranged from myotube targeting genes such as *perdido* (*kon-tiki*) [*48*] to key components of the sarcomere such as myosins, actins and troponins. This contrasts with a control group of genes classified with GO terms such as ‘Intracellular’, ‘Extracellular’, and ‘Mitochondrion’ which showed no enrichment in ‘Embryo Lethal’, ‘Pupal Lethal’, ‘Climbing defect’ or ‘All hit’ classes but was enriched in the ‘None’ class (Figure 3B).

### Identification of genes required specifically for adult muscle maintenance

**Figure 4:**
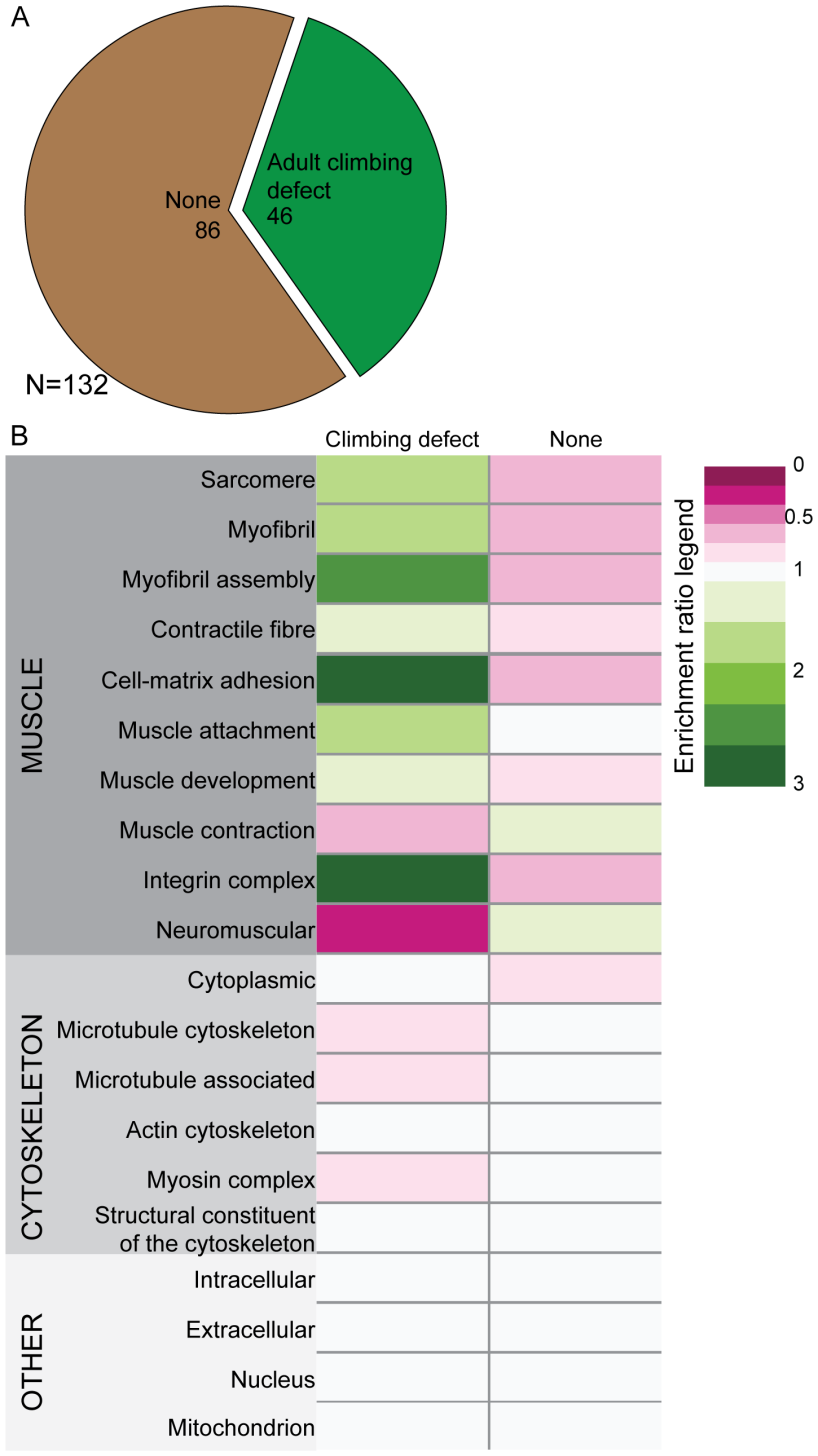
Results of the TARGET Screen for adult muscle maintenance. (A) Phenotypic breakdown of results from the TARGET screen. In the TARGET screen, RNAi constructs were only expressed after flies had eclosed. We identified 46 genes that caused climbing defects in adults as assayed by a negative geotaxis assay. The remainder, 86 genes, had no identifiable climbing defect. (B) As in Figure 3, we performed a GO term enrichment analysis by phenotype. GO terms are listed on the left. Note the enrichment in sarcomere-associated terms as well as “integrin complex”. Enrichment ratios were calculated by comparing frequency of a term in a specific phenotypic class to the frequency of the same term in the entire screened set. An enrichment ratio of 0 indicates that a given term did not appear in the phenotypic group. An enrichment ratio of <1 indicates that the frequency for the term was reduced in the phenotypic class compared to the whole screened set. An enrichment ratio of 1 indicates that the frequency for the term was the same in the phenotypic class compared to the whole screened set. An enrichment ratio of >1 indicates that the frequency for the term was enriched in the phenotypic class compared to the whole screened set.

Our screen identified genes specifically required for adult muscle maintenance using the TARGET system to limit RNAi expression to adults. The 132 genes identified in the Mef2:GAL4 screen whose knockdown resulted in a phenotype were screened in the TARGET screen [42]. The TARGET screen identified 46 genes, whose knockdown caused a significant decline in climbing ability in adult flies compared to control flies (Figure 4A). Forty of these genes had no previously known role in muscle maintenance. The remaining 86 genes had no phenotype when knocked-down in adult muscles (Figure 4A). Analysis of GO term distribution showed that muscle-associated terms such as myofibril assembly, sarcomere, muscle attachment, and cell-matrix adhesion were enriched in the 46 candidate genes (Figure 4B). As in the Mef2:GAL4 screen, GO terms such as ‘Intracellular’, ‘Extracellular’, ‘Nucleus’, and ‘Mitochondrion’, showed no such enrichment.

**Figure 5:**
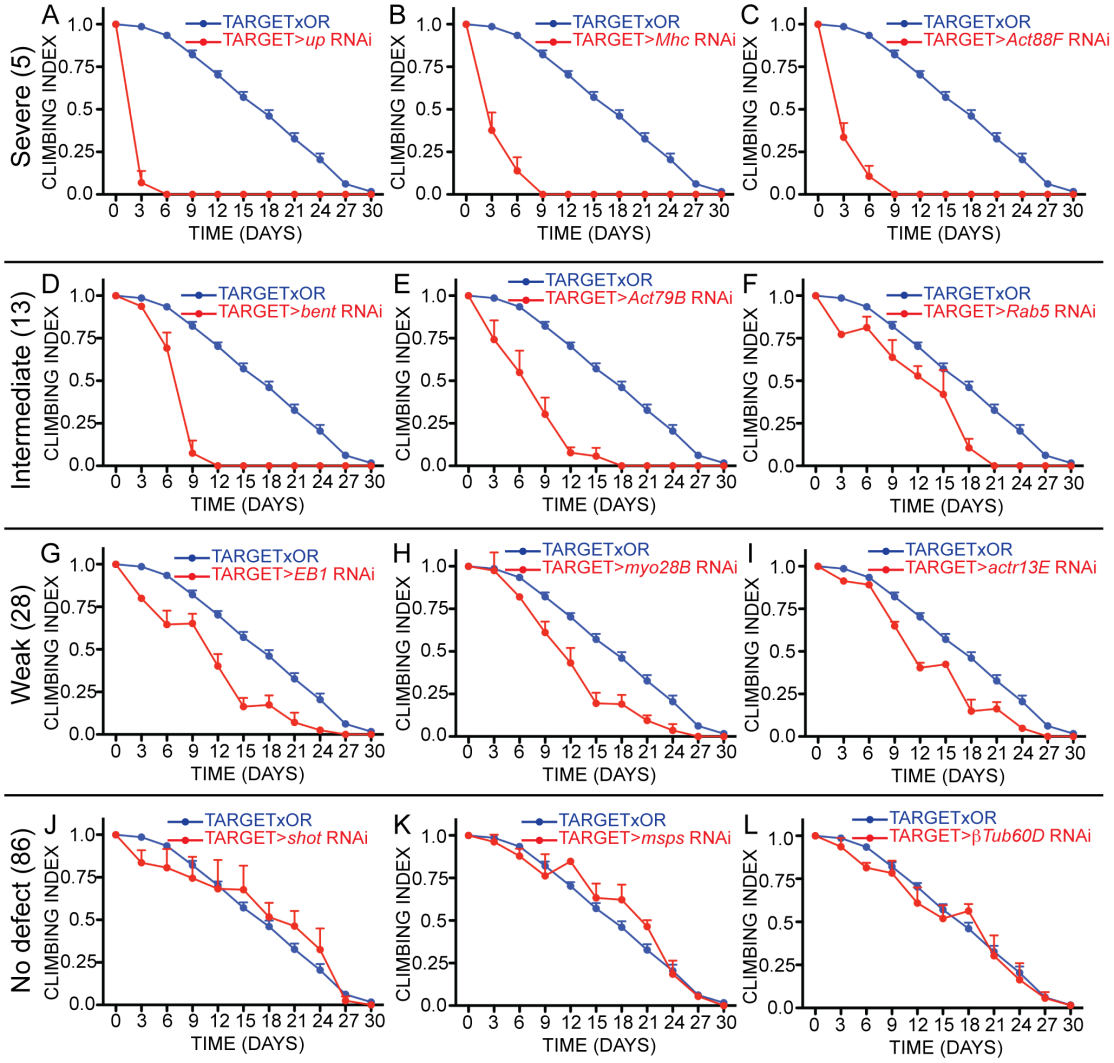
Sample assay graphs from TARGET screen. Severity of the climbing phenotypes identified in the TARGET screen were classified based on when flies lost the ability to climb. Loss of climbing ability between Day 0 and Day 9 were classified as ‘Severe’; between Day 12 and Day 21 were classified as ‘Intermediate’; between Day 24 and Day 30 were classified as ‘Weak’; and ‘None’ if climbing ability was not altered. Representative samples for each of the four classes were selected. (A-C) Examples of ‘Severe’ phenotypes. *up* RNAi (A), *Mhc* RNAi (B), and *Act88F* RNAi (C). In total, 5 genes had caused ‘Severe’ climbing defects. (D-F) Examples of ‘Intermediate’ phenotypes. *bent* RNAi (D), *act79B* RNAi (E) and *rab5* RNAi (F). In total, 13 genes caused ‘Intermediate’ phenotypes. (G-I) Examples of ‘Weak’ phenotypes. *EB1* RNAi (G), *myo28B* RNAi (H) and actr13E RNAi (I). In total, 28 genes caused Weak' phenotypes. J-L. Examples of ‘None’ phenotypes. *shot* RNAi (J), *msps* RNAi (K) and *βTub60D* RNAi (L). In total, 86 genes had no effect on adult climbing ability. For all graphs, blue lines are the control and red lines are the RNAi-mediated gene knockdown line. Error bars are standard error. For all lines, climbing ability was normalized to the first timepoint, Day 0, before RNAi construct expression was started.

**Figure 6:**
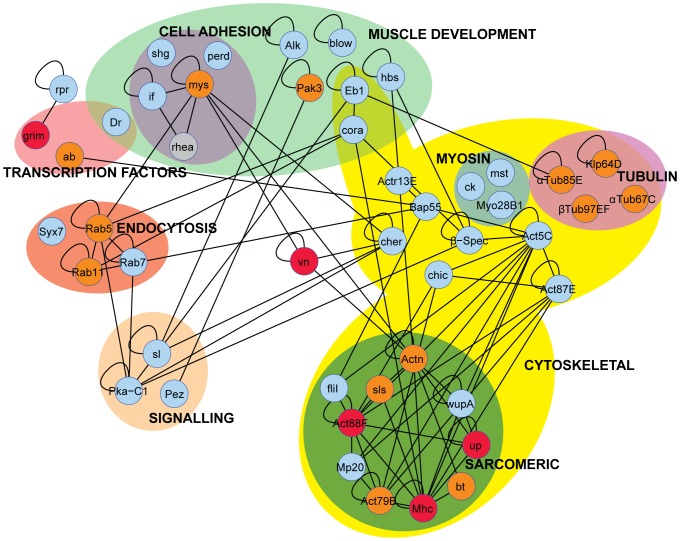
Genetic interaction network of TARGET screen hits. Genetic and biochemical interactions of the 46 genes identified in the TARGET screen were mined from the Drosophila Interactions Database (www.droidb.org). Lines linking each gene indicate direct genetic or protein-based interactions. Reflexive loops indicate a direct genetic or protein-based interaction with itself. Genes were then organized into functional groups based on GO term analysis. Each gene in the network was colour-coded based on the severity of the climbing phenotype caused by RNAi-mediated gene knockdown (see Figure 5). Red indicates a “Severe” phenotype; orange indicates a “Intermediate” phenotype; and blue indicates a “Weak” phenotype. The gene *rhea* is coloured gray as it was not part of this screen but was previously identified, using the same approach, to be required for muscle maintenance. Note cluster of genes that caused “Severe” or “Intermediate” phenotypes in the ‘Sarcomeric’ group.

The severity of climbing defects caused by the gene knockdown varied widely. We classified the phenotypes as ‘Severe’, meaning complete loss of climbing ability within 9 days of RNAi induction (5 genes; Figure 5A-C; Figure 6, red circles); ‘Intermediate’, meaning complete loss of climbing ability after 12 to 21 days of RNAi induction (13 genes; Figure 5D-F; Figure 6, orange circles) and ‘Weak’, meaning complete loss of climbing ability did not appear until more than 24 days after RNAi induction (28 genes; Figure 5G-I; Figure 6, blue circles). The remaining 86 genes, the class ‘None’, showed no difference from the control flies in climbing ability (Figure 5J-L). In the phenotypic classes ‘Severe’ or ‘Intermediate’, lethality typically followed the loss of climbing ability. The ‘Weak’ class was the largest by far, highlighting the value of our sensitive approach to identify more subtle defects. Figure 5 shows examples of the climbing ability decline for the four phenotypic classes. These were selected as the most representative examples in each of the phenotypic classes. The graphs for *bent*, *Mhc* and *Act88F* were included to demonstrate the severity of their phenotypes for context with the more detailed analysis we performed. Lethality without of a significant loss in climbing ability was not observed in the TARGET screen. Overall, our screen identified a set of 46 genes whose ongoing synthesis is required for maintenance of adult muscle function.

### Network analysis of genes identified in TARGET screen reveals key complexes in muscle maintenance

To assemble a network of candidate hits from the screen a two-step approach was utilized. Firstly, the Drosophila Interactions Database (www.droidb.org) was used to identify genetic and biochemical interactions between the 46 genes shown in the TARGET screen to be required for muscle maintenance (Figure 6). Secondly, GO term analysis was used to group the 46 genes into broad functional categories in order to further arrange the interaction network (Figure 6). This approach highlighted the importance of the network of interacting sarcomeric protein in muscle maintenance (Figure 6) as highlighted by the clustering of severe climbing phenotypes (red and orange circles) for this group. In particular, the Z-disc component α-actinin (*actn*) appeared as an important link between the ‘Sarcomeric’ interaction cluster and other clusters in the network. One such cluster that links to α-actinin is the Integrin Adhesion Complex (IAC), which our previous work has shown is important for muscle maintenance [27]. We previously identified the gene *rhea*, which encodes the IAC component Talin, to have a key role in muscle maintenance and have included it here to show the importance of the IAC [27]. Interestingly, about a quarter of the genes identified in the TARGET screen are associated with the GO term ‘Muscle Development’, and over half of the genes in this category have not been previously assigned a role in muscle maintenance. Several signaling molecules were also in our list. Some, such as PKA-C1 and *sl* (PLC-**γ**), interact with genes in the ‘Cytoskeleton’ and ‘Muscle Development’ clusters. Thus, our analysis identified key complexes of genes whose ongoing transcription and renewal is essential to maintain muscle function.

### RNAi constructs targeting *bent*, *Mhc* or *Act88F* causes significant reductions in mRNA levels

**Figure 7:**
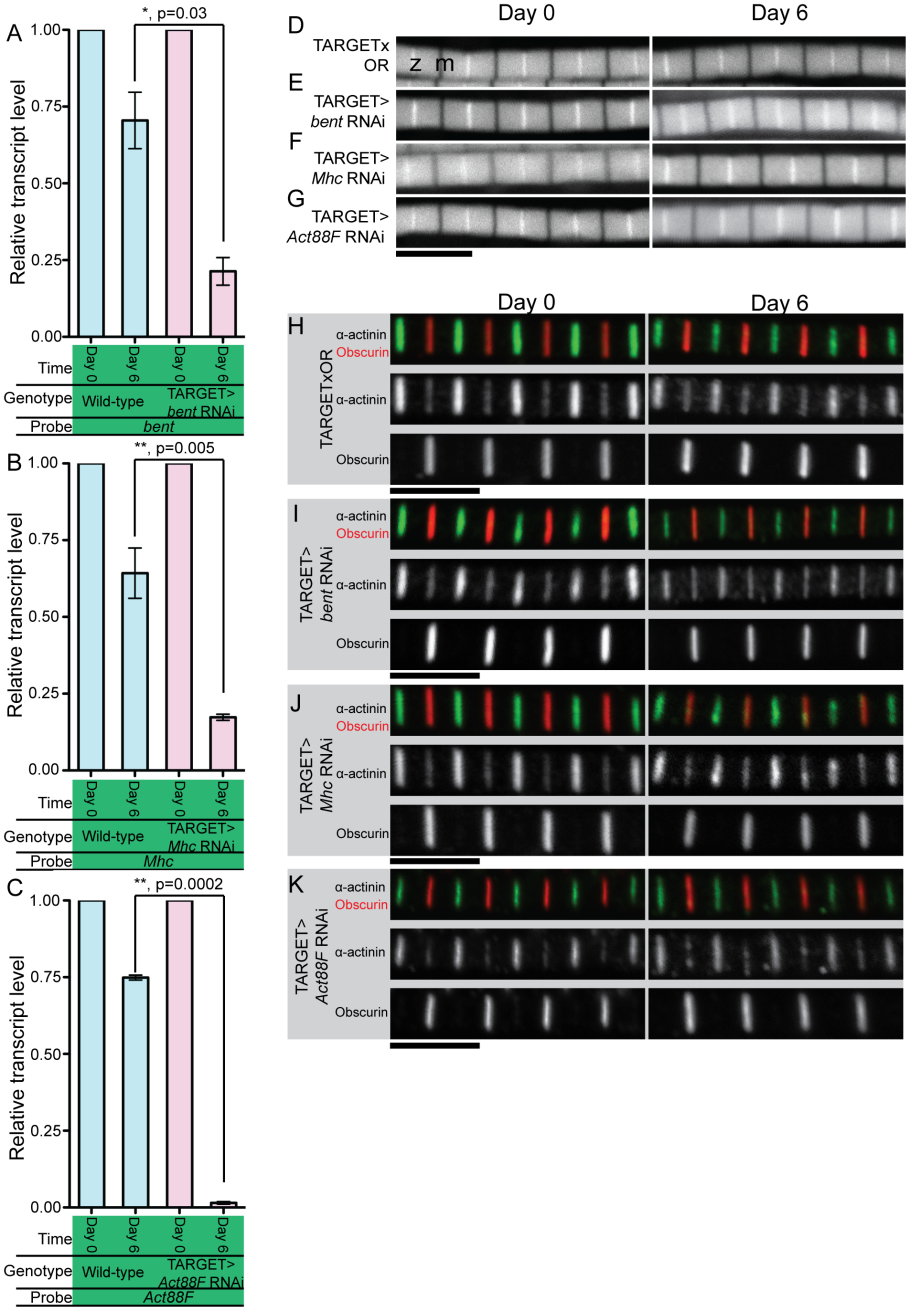
Loss of *bent*, *Mhc* or *Act88F* does not disrupt sarcomeric architecture. Using phalloidin and antibody labelling, IFM integrity was examined before and after *bent*, *Mhc* or *Act88F* RNAi expression. (A-C) qPCR analysis confirmed that expression of RNAi constructs targeting *bent*, *Mhc* or *Act88F* leads to substantial reduction in transcript levels for all three genes compared to control flies of equivalent age. For (A-C), 3 independent RNA extractions using 10 flies for each time-point were performed. GAPDH was used as an internal expression control. (D-G) Phalloidin stainings of sarcomeric F-actin before and after RNAi expression showed no defects in thin filament organization after 6 days of RNAi construct expression for all three genes. Z-discs are bright lines and M-lines are dark lines as indicated by the labels ‘z’ and ‘m’ in the TARGET>OR Day 0 image. (H-K) To examine the integrity of key sarcomeric structures, we used antibodies to label the Z-disc and the M-line. Z-discs were labelled with an α-actinin antibody (green) and M-lines were labelled with an Obscurin antibody (red). No disruption to the integrity of either structure or to sarcomeric actin was observed for any of the three genes. For all panels, scale bars are given below all images in black and are 5 µm; error bars indicate standard error; n.s. indicates not significant; * indicates a p-value<0.05, ** indicates a p-value<0.005, *** indicates a p-value<0.0005.

In our network analysis of genes required to maintain muscle maintenance the largest group was the ‘Sarcomeric’ cluster. Thus, based on the severity of the functional defect caused by their knockdown (Figure 5), their importance to sarcomeric structure as shown, in part, by the network analysis (Figure 6), three candidate genes were selected for further analysis: *bent*, *Mhc*, and *Act88F*. *bent* encodes Projectin, which fulfills part of Titin's role in the sarcomere and provides elasticity and structural support by linking thick filaments to the Z-disc [12]. *Mhc* encodes Myosin Heavy Chain, which forms the thick filaments and binds to actin in a cyclical manner to generate contraction [10]. Finally, *Act88F* codes for Actin88F, the IFM specific sarcomeric actin in the fly [10]. These three genes are, typically, the most abundant proteins in the sarcomere and loss of any of them during development causes defects in sarcomeric structure [7], [12], [21], [44]. We confirmed, using qPCR, that the RNAi lines we used gave effective knockdown in the levels of all three genes (Figure 7A-C). Although in control flies there is a slight reduction in the level of these sarcomeric components over the first days of life the expression of the RNAi gave rise to significant depletion in mRNA levels compared to the control (Figure 7A-C). *bent* mRNA levels were reduced by 79% over 6 days of RNAi expression compared to a 30% reduction in control flies of the same age (Figure 7A). *Mhc* mRNA levels were reduced by 83% over 6 days of RNAi expression compared to a 36% reduction in control flies of the same age (Figure 7B). *Act88F* mRNA levels were reduced by 98% over 6 days of RNAi expression compared to only a 25% reduction in control flies of the same age (Figure 7C). mRNA levels were assayed at these time-points due to the early lethality caused by RNAi induction. Since RNA was extracted from whole flies but the knockdown was carried out only in muscles these results may underestimate the extent of the knockdown and indicate that it is effective.

### Sarcomeric architecture is maintained after knockdown of *bent*, *Mhc* or *Act88F* in adult flies

**Figure 8:**
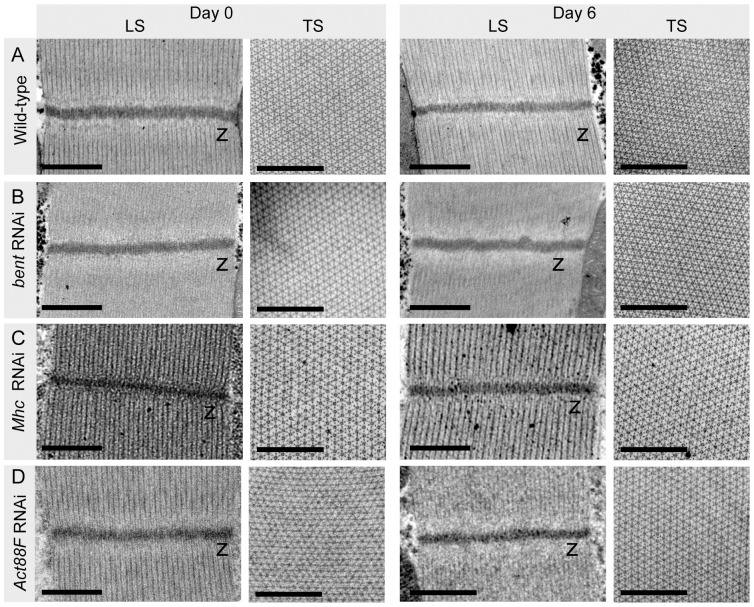
Loss of *bent*, *Mhc* or *Act88F* does not disrupt sarcomeric ultrastructure. We more closely examined the Z-disc and filament lattice integrity in *bent*, *Mhc*, and *Act88F* RNAi flies using transmission electron microscopy. The IFMs from *bent*, *Mhc* and *Act88F* knockdown flies were imaged before RNAi expression (Day 0) and again after 6 days. (A-D) Electron micrographs of Longitudinal Sections and Transverse Sections of IFM sarcomeres before and after RNAi expression showing no defects to Z-disc integrity or myosin filament lattice architecture. For LS: ‘z’ indicates Z-discs; for TS: larger circles are myosin, smaller dots are actin. For all panels, scale bars are overlayed in black and are 500 nm. See Figure 1A for schematic representations of longitudinal and transverse sections of IFMs.

To determine whether the functional defects observed upon knockdown of *bent*, *Mhc* and *Act88F* were due to degeneration of the sarcomeres, sarcomeric architecture was analyzed using confocal microscopy and immunohistochemistry. IFMs were examined before and after 6 days of RNAi construct expression. Surprisingly, no obvious defects were observed in *bent*, *Mhc* and *Act88F* RNAi flies. The distribution and organization of F-actin, as labelled by phalloidin, remained intact throughout the 6 day time course in RNAi-mediated knockdown flies (Figure 7D-G). The integrity of the Z-disc and M-line was examined using antibodies to label the Z-disc (α-actinin [49]) and M-line (Obscurin [11]) (Figure 7H-K). The RNAi-mediated knockdown of *bent*, *Mhc*, or *Act88F* did not result in defects to the Z-disc or the M-line compared to the control flies (Figure 7H-K). Taken together with the severe functional defects observed for these three genes (Figure 5B-D), the lack of obvious defects in sarcomeric actin organization or Z-disc and M-line integrity suggests that sarcomeric structure may be disrupted in a subtler way. To address this possibility we used Transmission Electron Microscopy (TEM) to more closely study the ultrastructure of IFM sarcomeres (Figure 8). As before, the RNAi constructs were expressed for 6 days. IFMs were imaged before and after RNAi expression. For all three genes no disruptions to Z-disc integrity (Longitudinal Sections) or filament lattice architecture (Transverse Sections) were observed (Figure 8A-D).

### *bent*, *Mhc* and *Act88F* are required to maintain sarcomere length in adult flies

**Figure 9:**
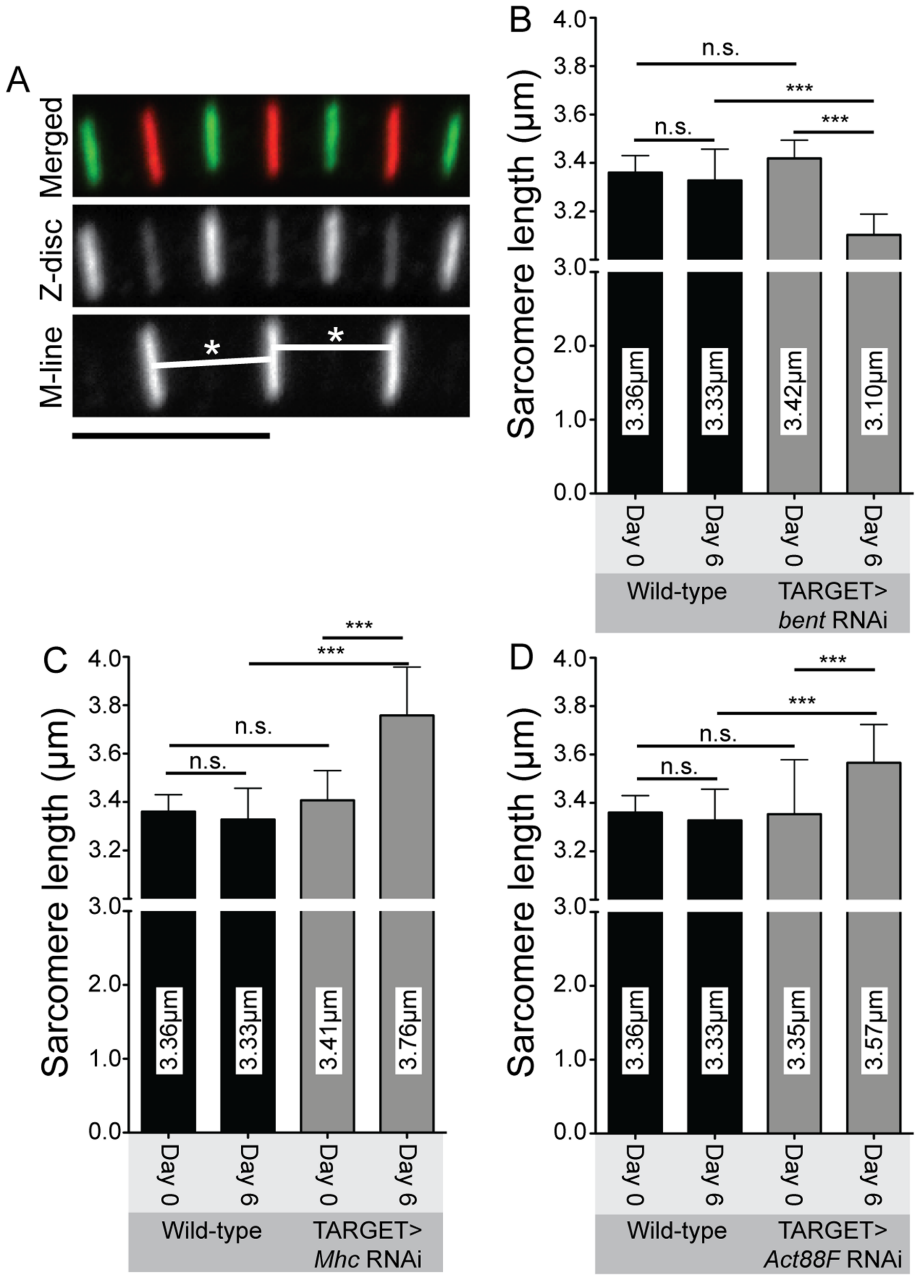
*bent*, *Mhc* and *Act88F* are required to maintain correct sarcomere length. Sarcomere length was measured from M-line to M-line using the Obscurin antibody as this provided a more precise definition of sarcomere length compared to the Z-disc antibody. For all three genes, sarcomere length was measured before RNAi construct induction and after six days of RNAi construct expression. (A) Schematic showing how sarcomere length was measured. Length was measured from the centre of each M-line to the centre of the adjacent M-line in straight myofibrils. White lines marked by asterisks indicate the measured distance of two adjacent sarcomeres. Scale bar is in black, 5 µm. (B) Sarcomere length for control and *bent* RNAi flies. Loss of *bent* caused sarcomeres to shorten significantly compared to those of control flies which, by contrast, showed no difference over the same time course. (C) Sarcomere length for control and *Mhc* RNAi flies. Loss of *Mhc* causes sarcomeres to lengthen significantly after six days of RNAi construct expression. The length of sarcomeres in the control flies does not change. (D) Sarcomere length for control and *Act88F* RNAi flies. Loss of *Act88F* causes sarcomeres to lengthen significantly after six days of RNAi construct expression. The length of sarcomeres in the control flies does not change. Representative images of Obscurin stainings can be found in Figure 7H-K. For B-D error bars indicate standard deviation; ns indicates a p-value>0.05, *** indicates a p-value = <0.0005. For B-D, N≥30 sarcomeres from 5 animals at each time-point.

Sarcomere length must be maintained due to its key role in muscle function [50]–[53]. To determine whether the loss of *bent*, *Mhc* or *Act88F* disrupted sarcomere length, we examined sarcomere length using confocal microscopy in *bent*, *Mhc* or *Act88F* knockdown flies before and after RNAi induction. Sarcomere length was then measured from M-line to M-line using an antibody targeting the M-line specific protein Obscurin (see Materials and Methods; Figure 9A). Over the 6 day time course control sarcomeres were observed to decrease from 3.36µm to 3.33µm, a 1% decrease in length, although this change was not statistically significant (Figure 9B-D). These wild-type sarcomere lengths were in line with previous reports [54]. However, in *bent* knockdown flies, sarcomeres were observed to significantly decrease in length over the same time period from 3.42µm to 3.10µm, a 9.2% decrease (Figure 9B). This change resulted in sarcomeres significantly shorter than the control ones. Conversely, knockdown of both *Mhc* and *Act88F* caused a significant increase length (Figure 9C, D). Loss of *Mhc* caused an increase in length from 3.41µm to 3.76µm, a 10.3% increase (Figure 9C). Loss of Act88F caused an increase in length from 3.35µm to 3.57µm, an increase of 6.3% (Figure 9D). In both cases the final length after 6 days of RNAi construct expression was significantly longer than the control sarcomeres. Overall, this data shows that knockdown of *bent*, *Mhc* or *Act88F* leads to defects in the maintenance of sarcomere length in the adult muscle.

### Disruption of sarcomere length in *bent*, *Mhc*, and *Act88F* flies is specifically due to changes in thin filament length

**Figure 10:**
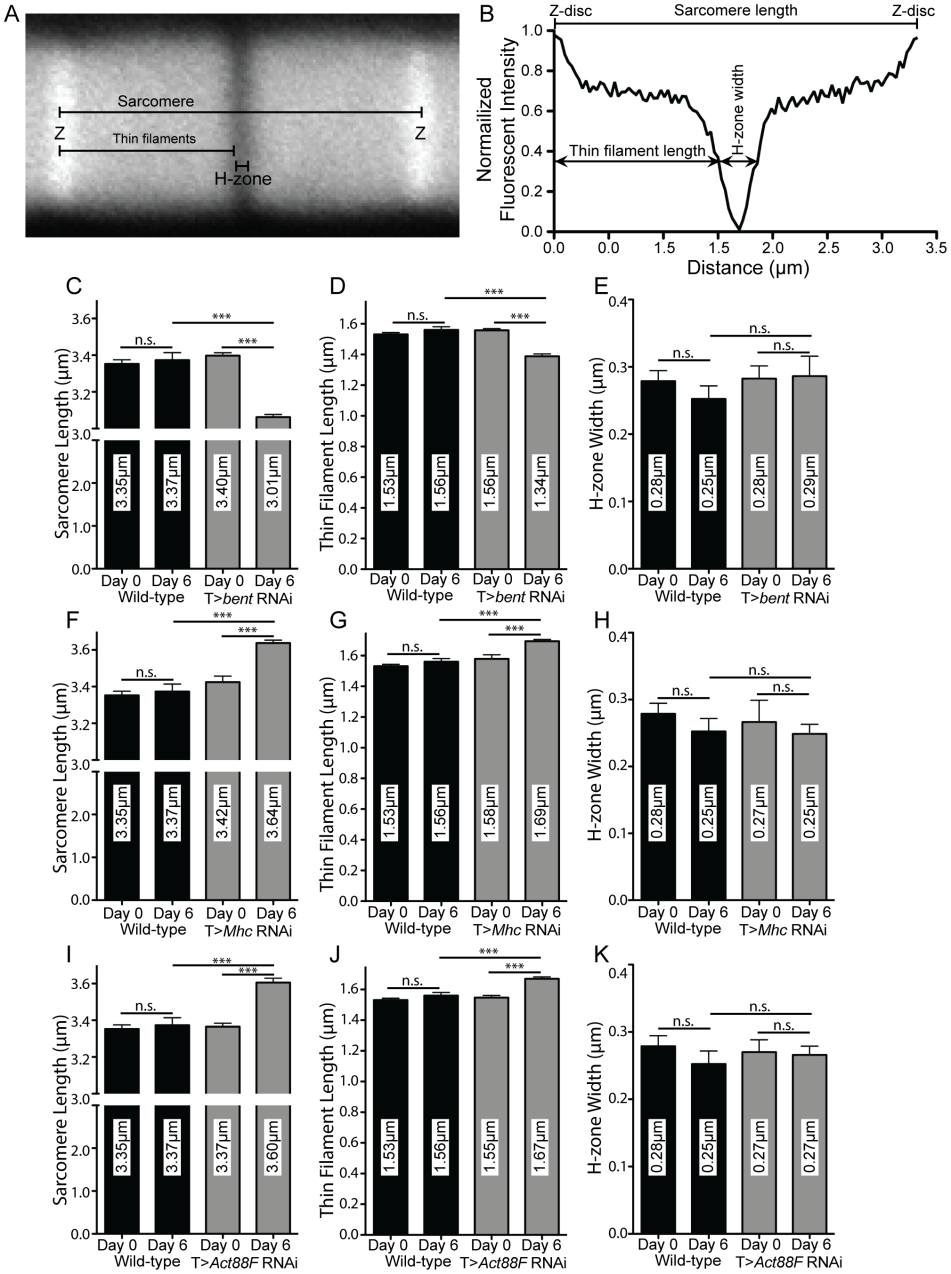
Sarcomere length defects are due to disruption of thin filament length. Distribution of actin across the sarcomere was used to determine sarcomere length, thin filament length and H-zone width. (A) Sample image of a single sarcomere labeled with phalloidin to show actin filaments. Black overlay lines indicate quantified parameters. (B) Sample profile of a sarcomere showing the normalized fluorescent intensities of pixels found along the overlay line labeled ‘Sarcomere’ in A. Measured parameters, “Sarcomere Length”, “Thin Filament Length” and “H-zone Width”, are indicated. (C-E) Quantification of Sarcomere Length, Thin Filament Length, and H-zone width respectively in control and *bent* RNAi construct-expressing flies. Sarcomere and thin filament length does not significantly change in the control flies, but significantly shortens in the *bent* RNAi construct-expressing flies over six days. (F-H) Quantification of Sarcomere Length, Thin Filament Length, and H-zone width respectively in control and *Mhc* RNAi construct-expressing flies. Sarcomere and thin filament does not significantly change in the control flies, but significantly increases in the *Mhc* RNAi construct-expressing flies over six days. (I-K) Quantification of Sarcomere Length, Thin Filament Length, and H-zone width respectively in control and *Mhc* RNAi construct-expressing flies. Sarcomere and thin filament length does not significantly change in the control flies, but significantly increases in the *Mhc* RNAi construct-expressing flies over six days. Representative images of phalloidin stainings can be found in Figure 7D-G For all panels, error bars indicate standard error; n.s. indicates a p-value>0.05, *** indicates a p-value<0.0005. For (C-K) N≥30 sarcomeres from 5 animals at each time-point.

To more closely interrogate how the loss of *bent, Mhc,* or *Act88F* disrupts sarcomere length, we analyzed the distribution of actin across the sarcomere by staining IFMs with phalloidin (Figure 10A). Phalloidin labels actin filaments and the Z-disc. As no filamentous actin is present in the H-zone, this structure is evident by the absence of phalloidin staining. Measuring the pixel intensity of a line across the phalloidin-labelled sarcomeres in a muscle fiber allows the determination of the thin filament length, H-zone width, and overall sarcomere length (Figure 10A-B; see Materials and Methods). This approach was used to analyze sarcomeres in *bent, Mhc* and *Act88F* knockdown flies before and after 6 days of RNAi induction. As observed in Figure 9, sarcomere length was reduced in the *bent* RNAi flies over the 6 day time period while increasing in *Mhc* and *Act88F* RNAi flies (Figure 10C, F, I). Although the results in Figure 9 were obtained by measuring the distance between adjacent M-lines using antibody labeling and Figure 10 measured Z-disc to Z-disc distance using phalloidin labeling, strikingly consistent lengths were obtained between the two experiments. Over the same time course the sarcomeres of control flies showed no significant change. We then examined the thin filament length and H-zone width, interpreted as the distance between the two thin filament arrays, to determine which parts of the sarcomeric anatomy were disrupted in order to cause the overall defects in length. For all three genes, no significant defects were observed in H-zone width (Figure 10E, H, K). However, for *bent* RNAi construct-expressing flies, a significant decrease in thin filament length was observed over 6 days (Figure 10D). Similarly, for *Mhc* and *Act88F* RNAi construct-expressing flies, thin filament length increased significantly over 6 days (Figure 10G, J). Our measurements of thin filament length were consistent with previously reported lengths for IFM sarcomeres of similar age [54]. These results show that the overall disruption of sarcomere length observed upon *bent, Mhc,* or *Act88F* RNAi-mediated knockdown in adult fly muscles is due to an incorrect regulation of thin filament length, and not to defects in H-zone width.

## Discussion

Animal muscles are a remarkably robust tissue, maintaining their structure and function through ongoing cycles of contraction and relaxation. It is their ability to carry out this function reliably that underlies animal locomotion. However, there are still many questions about how muscles maintain their ability to function over the life of an organism. We have gained insight into this process through a genetic screen designed to take advantage of the amenability of fly muscles to large-scale genetic analysis [42]. Previous large-scale screens, both *in vivo* and in primary cell culture, have established the fly as an excellent system in which to analyze muscle function [41] as well as specific aspects of muscle development such as myoblast fusion [55], myotube targeting [48], and muscle assembly [56]. Furthermore, these screens identified a large number of genes required for muscle function [41], [56]–[58]. Unlike these studies, our screen focused on the adult fly by limiting gene knockdown to flies that had completed myogenesis. The advantage of our approach is that we can identify those genes that are specifically required for ongoing muscle function with a statistically robust analysis. The disadvantage of our approach is that it is labour-intensive and time-consuming. For this reason we used a candidate gene approach to limit our screen to a list of 238 genes.

The cytoskeleton is the major component of the sarcomere and, as such, we hypothesised that genes whose ongoing expression is required to maintain muscle function would be highly represented in this category. Thus, we focused our screen on the cytoskeleton. A comprehensive bioinformatic analysis identified the complete set of genes that make of the fly “cytoskeletome” [59]. We obtained all available RNAi constructs targeting components of the cytoskeleton from several RNAi stock centers. Additionally we performed a literature review to identify genes required for muscle function and included as many of these as possible. Finally, we included a negative control set of genes that had no known function in muscles [42]. By combining the tools of genomics with a more targeted approach our screen identified a good number of genes (46 out of 238 or 19.3%), most of which had not been previously assigned a role in muscle maintenance. Importantly, only one of the genes, *Rab7*, was previously identified in the very comprehensive genome-wide screen [41] as being required for muscle function in the adult. This illustrates the usefulness and novelty of our approach. It is important to note that the screen we performed to identify genes required for muscle maintenance used leg muscle function as a read-out [42]. This caveat should be kept in mind while interpreting the results presented in our study as we use the indirect flight muscle to study the morphology of the muscle. While the indirect flight muscle is a highly specialized muscle, we nonetheless believe that it is likely that the underlying mechanisms of muscle maintenance are conserved as IFM retains a high degree of similarity to the other skeletal muscles in the fly.

As a complimentary approach to the identification of genes whose knockdown in the adult results in failure to maintain muscle function, we characterized the transcription of sarcomeric components as well as the turnover of sarcomeric actin. While it has long been known that muscles are highly dynamic during myogenesis – when they undergo cell fusion, migration, cellular rearrangement, and sarcomeric cytoskeleton assembly – we show that they continue to be dynamic throughout adulthood. Furthermore, we demonstrate that sarcomeric actin undergoes protein turnover in the adult sarcomere, primarily at the Z-disc. Through our screen the model of adult muscle that emerges is one of a tissue in a constant state of flux and renewal. Moreover, our work shows that turnover of a key subset of cytoskeletal proteins underlies long-term muscle maintenance.

Previous work has shown that muscle architecture, in particular cytoskeletal architecture, becomes disorganized in aged flies (∼50 days or older) [43]. However, it was not previously known whether, and to what extent, muscles in younger flies – within the first few weeks of life – changed with time. Here, we illustrate that over the first 30 days of fly life muscles undergo subtle changes that involve synthesis of new sarcomeric components that are then incorporated into the muscle. This result is consistent with previous work showing that sarcomeric components were expressed in adult fly muscles [60]. Our data is also in line with the body of literature illustrating protein turnover in sarcomeres of cultured mouse myocytes [61], cultured chick cardiac muscles [62], rat skeletal muscles [62], [63], and the body wall muscles of adult nematodes [64]. Nonetheless, our study is unique because it follows the turnover of sarcomeric actin over many days in intact animals and at multiple time points. In particular, our analysis reveals that sarcomeric actin is rapidly incorporated into the muscle and remains there for a period of days. Moreover, we observed that the majority of the actin incorporates in the sarcomere at the Z-disc. This finding is in line with previous results suggesting that the Z-disc is a dynamic structure [65], [66] as well as with the hypothesis that the Z-disc serves as the initial point of actin incorporation into sarcomeres [67]. The notion that the Z-disc acts as a site for actin incorporation into the sarcomere is particularly appealing as the barbed-end of sarcomeric actin filaments are located in the Z-disc and actin monomers are preferentially added to the barbed-end during polymerization [53]. We also observed Act88F::GFP in the M-line. This could potentially be the result of pointed end incorporation of into the thin filament [67]–[69]. Our *in vivo* analysis of actin turnover rates showed that turnover occurred over dozens of hours to days, a result consistent with previous studies in rat and mouse myocytes [61], [63]. In summary, we demonstrated that synthesis and turnover of core sarcomeric proteins is a feature of adult fly muscles and that the Z-disc is the initial point of incorporation for new actin.

The mechanism underlying the non-specific incorporation of eGFP into the sarcomere observed in Figure 2 is unclear. However, we feel that the following scenario is likely. eGFP is produced in abundance by, both the Mef2:GAL4 system and the TARGET system, and saturates the sarcoplasm. As a relatively small molecule, it is able to diffuse throughout the myofibril. The Z-disc is the primary location of protein incorporation into the sarcomere, and we speculate that more eGFP is able to enter here. Alternatively, the structure of the Z-disc might preferentially accumulate eGFP as it is less dense than sarcomere body. Furthermore, eGFP intensity does not decline with time, suggesting that there is no active mechanism in place to remove it from the myofibril as there is for Act88F::GFP. Our data indicate that the incorporation of eGFP into the sarcomere is a non-specific process while the incorporation of Act88F::GFP is regulated. This explains the lower fluorescent intensity in the TARGET>Act88F::GFP sarcomeres compared the Mef2:GAL4>Act88F::GFP sarcomeres. Newly synthesized Act88F::GFP in the pulse-chase experiment must compete for incorporation into the sarcomere with unlabeled endogenous copies.

Given our results, which support the idea that adult muscles are a dynamic system, it is perhaps not surprising that we identified many genes whose ongoing expression is required to maintain muscle function. Nonetheless, a key aspect of our screen was the high-resolution, statistically robust assays we performed. Had we assayed muscle function only once, a week after flies had eclosed as was previously done [41], rather than every 3 days and in quintuplicate, we would have missed a substantial number of candidate hits. In this respect our screen represents a compromise between genome-wide low-resolution/high-throughput studies and high-resolution/low-throughput analyses of single genes. Moreover, our high-resolution approach is necessitated by the observation that wild-type flies exhibit a natural decline in muscle function over the first thirty days of life immediately following eclosion [17], [27], [70]. This means that in effect we were screening for genes that enhanced a decline that already exists in the wild-type, a somewhat subtle phenotype. Thus it was important that we carefully controlled the experimental conditions as environmental changes such as temperature, humidity [71], lack of exercise [17] and overcrowding can quicken the natural decline in climbing ability.

Our preliminary screen using the Mef2:GAL4 system to express the RNAi constructs throughout muscle development, was designed to identify RNAi lines whose expression gave rise to phenotypes during muscle development as well as adulthood. Furthermore, it allowed us to draw a number of interesting conclusions. Firstly, 89% of the genes we screened in the Mef2:GAL4 screen were previously screened in the genome wide RNAi screen [41]; of these, the expression of 82% gave rise to the same phenotypes in the two screens – a high degree of overlap that validates both screens. Secondly, compared to a control group of miscellaneous genes we observed a higher hit rate for the group composed of genes that are classified under GO terms in the cytoskeletal and muscle category. Unsurprisingly, we saw strong enrichment in our list of candidate genes for GO terms in myofibril structure and development categories, especially in the more severe lethal phenotypic group ‘Embryo lethal’. Such genes included components of the Integrin Adhesion Complex, myosin subunits, Actin subunits and other structural components of the sarcomere such as Projectin (*bent*) and *sallimus*. This underscores the importance of screening such genes by limiting RNAi knockdown in the adult, as we did in the TARGET screen. Finally, although GO terms for actin, myosin, and their respective binding partners were enriched in the Mef2:GAL4 screen, genes annotated with microtubule-associated GO terms, tubulin subunits and related microtubule motors, were largely absent from our list of candidates for all phenotypic groups. This may suggest that microtubule dynamics serve a more restricted role in muscle development, or that a small core of microtubule-associated proteins mediates such roles. In summary, our results highlight the importance of the actin and myosin cytoskeleton in myogenesis.

Our two-step screening protocol enabled us to eliminate half of the total set of genes from the more labor-intensive TARGET screen. Of the 132 genes we screened using the TARGET system only 46 (35%) were identified as being required for adult muscle maintenance though all gave phenotypes when knocked-down throughout muscle development with Mef2:GAL4 [42]. This suggests that fewer genes are required for maintaining muscles than are required for building muscles. Interestingly, 22 genes that gave an adult climbing defect when knocked down with Mef2:GAL4 did not do so with the TARGET system, suggesting that the adult phenotypes are a result of abnormal muscle development and supporting our assertion the genes identified in the TARGET represent a unique set. GO term analysis of the candidates identified in the TARGET screen provided some intriguing insights into the comparative roles of genes in muscle development and maintenance. Unsurprisingly, genes annotated with myofibrillar GO terms were enriched in the set of genes that are required for muscle maintenance as were components of the integrin adhesion complex, which is consistent with our previous results [27]. There is therefore substantial overlap in the set of genes that make muscle and those that maintain it. Moreover, network analysis of the candidate genes reveals clusters of interacting genes, likely complexes, which are important for maintaining muscle function. While the most prominent of those is, as expected, the sarcomeric cluster of proteins, a number of other clusters are identifiable including the endosomal cluster of Rab11/Rab5/Rab7. Importantly the network analysis helps identify genes that serve an important function in linking different clusters, most prominently the genes *actn* (α-actinin) and *cora* (coracle).

Although our screen was designed to identify subtle phenotypes it is intriguing that robust functional defects were not associated with greater substantial disruptions of muscle architecture and morphology for many of our candidate genes. One potential explanation is that the half-life of sarcomeric components is much longer than for other proteins, indeed our turnover data supports this idea, and that severe morphological defects do not appear until later. This suggests that functional defects can occur prior to the appearance of substantial morphological defects and that muscles can appear to be healthy while being functionally compromised. Another possibility is that the muscle is able to sense when the pool of newly synthesized proteins is depleted and delay the removal of proteins from the sarcomere even if they have been damaged. Thus we do not expect that substantial protein depletion from the sarcomere is the root cause of the functional defects we observe.

When 3 candidate genes (*bent*, *Act88F*, *Mhc*) were chosen and analyzed in greater detail a notable morphological defect was identified, namely a disruption in sarcomere length and more specifically thin filament length. We analysed sarcomere length in two different ways, Actin profiles and antibody stainings, and found highly reproducible disruptions in sarcomere length for all three analysed genes with both methods. While *bent* knockdown resulted in shortened sarcomeres, *Mhc* and *Act88F* knockdown caused an increase in sarcomere length. These differences might be due to the different roles that these proteins play within the sarcomere. Projectin, and its vertebrate homolog Titin, is involved in determining sarcomere length by acting as a sarcomeric ruler during development [53], [67]. Our results indicate that this role persists after development is complete. Given the known role of Titin/Projectin as a scaffold that links the Z-disc and the thick filaments we speculate that a reduction in Projectin levels could result in the disruption of this. This could lead to a collapse of the sarcomere upon itself, a similar state to that which exists in a contracted muscle. This speculation is in line with previous studies that implicated vertebrate Titin in maintaining sarcomeric architecture [72]. Alternatively, it is possible that the shortening of thin filaments observed in *bent* knockdown flies could be a compensatory mechanism to preserve sarcomere functionality. It has been proposed that sarcomere shortening could act as a protective adaptation in weakened muscles as shorter sarcomeres generate less force and thus subject themselves to less mechanical strain [25], [73]. Myosin works with Projectin/Titin as part of the sarcomeric scaffold to regulate thin filament length. In *Mhc* null flies, thin filament length is highly variable suggesting that Myosin is required to specify thin filament length [74]. The abnormal thin filament lengths we observed in the *Mhc* knockdown flies suggest that the role of myosin in determining thin filament length is as important during muscle maintenance as it is in muscle development. In general, our data underscore the importance of maintaining proper sarcomere dimensions throughout the life of the organism and highlight the role of protein turnover in this process. Indeed, disruptions to thin filament length are frequently associated with a range of muscular diseases [53].

We suspect that failure to maintain proper sarcomere integrity underlies the functional phenotypes observed in at least some of the 44 other candidate genes although there are other alternatives. For example, a subset of the candidate genes may affect neuronal function, potentially by having an essential role in the NMJ; such candidates include *abrupt* and the Rab genes [75]–[78]. Another possibility is that the decline in climbing ability is due to muscle-type specific phenotypes such as defects in the fly heart muscle rather than a general defect in muscle maintenance. However it is unlikely that core sarcomeric components such as actin, Projectin, and Mhc are required more in some muscle-types than others. Finally, one common pathway implicated in loss of muscle function in adult flies is mitochondrial degeneration [32], [33]. Mitochondrial GO terms show no enrichment in our set of candidate genes, suggesting that loss of mitochondrial function does not play a large role in the functional phenotypes observed in our screen. Nevertheless, our working hypothesis, that the observed functional defects induced by the knockdown of our candidate genes are caused by changes in sarcomere length, is in line with previous observations in the literature [25], [51], [79]. It is, for example, known that mutations in sarcomeric components such as Mhc and Troponin-T screen lead to changes in sarcomere length by causing muscle hypercontraction [24]. It should be noted that although the phenotypes we observe in *bent* knockdown flies are reminiscent of muscle hypercontraction mutants they differ somewhat. Hypercontraction typically refers to excessive contraction or underlying developmental defects that disrupt the regulation of contraction in adult flies [21], [24]. As defined previously [24] sarcomere shortening, and hypercontraction in particular, is associated with filament density disruption and other ultrastructural defects which we did not observe. We predict that further analysis of the 46 candidate genes we identified will reveal there is no universal underlying mechanism for muscle maintenance.

## Conclusion

The data presented here show that the ongoing synthesis and turnover of a subset of cytoskeletal proteins is essential for maintaining muscle function. Moreover, the Z-disc was identified as the initial site of protein turnover within the sarcomere. We propose, based on our observation of actin dynamics over multiple days in adult muscle, that the Z-disc provides an entry point for newly synthesized proteins, which then spread throughout the rest of the sarcomere. Failure to renew muscles through protein turnover results in accelerated functional decline compared to control flies. Since muscles experience continual stress this may be due to a failure to replace damaged proteins, allowing their accumulation and leading to a disruption of muscle function. This could explain why we did not see stronger ultrastructural defects when knockdown flies were examined; the protein might be present, preserving the ultrastructure, but their function could be compromised due to mechanical or oxidative damage. Consistent with this idea, the defects that were observed in thin filament and sarcomere length are reminiscent of, though not identical to, other conditions that disrupt the ability of the muscle to generate force, such as hypercontraction [24], [25], [51]. In addition it is intriguing to speculate that our screen provides a model for some of the functional decline associated with the process of aging. We hypothesize that aging-associated functional decline is due to a reduced ability of the muscle to replace damaged components. In line with this hypothesis studies on aging muscle in flies and humans show that aging is associated with a general decrease in the synthesis of proteins in the muscle [43], [80]. The genes we identified in our screen represent excellent candidates to study in the context of myopathy and aging. Furthermore, they are potential therapeutic targets in the treatment of myopathies characterized by accelerated, aging-associated loss of adult muscle function.

## Materials and Methods

### Fly stocks and genetics

All TARGET and RNAi-mediated gene knockdown experiments were performed using flies that expressed the short hairpin constructs under UAS control. Males homozygous for each UAS-RNAi line were crossed to virgin TARGET females of the genotype *P[tubP-GAL80ts]9/FM7;Mef2:GAL4/TM3*. The F1 progeny was raised at 18°C until eclosion at which point virgin females were collected and transferred to 29°C to induce RNAi expression. As a control for the RNAi-mediated knockdown experiments male wild-type Oregon-R flies were crossed to virgin females of the genotype *P[tubP-GAL80ts]9/FM7;Mef2:GAL4/TM3* and raised under the same conditions as the RNAi construct-expressing lines. The RNAi constructs were obtained from the Vienna Drosophila RNAi Center (VDRC, http://stockcenter.vdrc.at [81]). The RNAi line targeting *bent* was *w[1118]; UAS-bent IR* (VDRC ID 46253), the line targeting *Mhc* was *w[1118]; UAS-Mhc IR* (VDRC ID 105355), and the line targeting *Act88F* was *w[1118]; UAS-Act88F IR* (VDRC ID 9780).

The Actin turnover experiments were performed using a UAS-*Act88F* GFP fusion line (Bloomington ID #9253) [47] and a UAS-eGFP line (Bloomington ID #6874). To drive expression of the transgenes throughout development, males from the two fluorescent lines were crossed to virgin females with the genotype y1,w*;P[GAL4-Mef2.R]. The F1 progeny were raised at 25°C. For adult specific expression of the transgenes, males were crossed to virgin females with the genotype *P[tubP-GAL80ts]9/FM7;Mef2:GAL4/TM3.* F1 progeny were raised at 18°C until eclosion at which point they were switched to 29°C to express the fluorescently labeled transgene.

### Electron Microscopy

Fly muscles were prepared as follows. Thoraces were bisected and immediately placed in 2.5% glutaraldehyde, 1.6% paraformaldehyde with 0.1 M sodium cacodylate buffer for 2 hours at room temperature (RT) (EMS, Hatfield, PA). Thoraces were washed in 0.1 M sodium cacodylate for 4×15 minutes. Thoraces were post-fixed for 2 hours at RT in 1% osmium tetroxide in 0.1 M sodium cacodylate (EMS, Hatfield PA). Samples were dehydrated with an ethanol series and embedded in a 1:1 Spurr's and Embed812 mixture (EMS, Hatfield PA). Ultrathin silver-gold sections (85 nm thick) were cut using a Leica Ultramicrotome and 2 mm ultra 45° DiATOME diamond knife (Leica Microsystems, Richmond Hill, ON; DiATOME, Biel, Switzerland). Sections were mounted on 200 mesh copper grids (EMS, Hatfield PA) and were double stained with uranyl acetate and lead citrate for 4 minutes each. Electron micrographs were obtained using a transmission electron microscope (FEI Tecnai G2 Spirit, FEI, Hillsboro, OR).

### Immunohistochemistry and confocal microscopy

Antibody staining was performed as previously described [27] with modifications. Fly thoraces were bisected to expose the IFMs. Thoraces were then fixed in 4% paraformaldehyde in PBS for 20 minutes at RT, rinsed with fresh PBT 3x, incubated for 1 hour in PBT and blocked in 0.2% BSA for 1 hour at RT. Thoraces were incubated with primary antibodies overnight (ON) at 4°C, washed, blocked in BSA and incubated ON at 4°C with secondary antibodies. IFMs were dissected out and mounted on glass slides. For phalloidin staining, thoraces were fixed in 4% paraformaldehyde in PBS for 20 minutes at RT, rinsed with fresh PBS 3x, washed for 1 hour in PBS then incubated ON at 4°C. The primary antibodies used were anti-obscurin (pAb rabbit, 1:200, gift of Dr. Belinda Bullard) and anti-α-actinin (MAC276, rat mAb, 1:200, gift of Dr. Belinda Bullard) [23]. Phalloidin was AlexaFluor 488 or rhodamine-phalloidin (Invitrogen). All sample preparation for quantification was performed in parallel. Confocal images were obtained using an Olympus IX81/FV1000 microscope with 10x/0.40, 40x/1.30 oil and 60x/1.33 oil objectives. Images were processed with Adobe Photoshop and ImageJ.

### Quantification of sarcomeric parameters

Sarcomere length was calculated as follows. Confocal micrographs using the Obscurin antibody were prepared as detailed above. This antibody specifically labels the M-line of sarcomeres (Figure 7H) allowing for precise measurements of the M-line-to-M-line distance of IFM sarcomeres (Figure 9A). For each data point, a total of at least 30 sarcomeres from at least 5 different animals were counted. Samples were fixed in parallel to eliminate fixation artifact. Sarcomere length was measured using ImageJ. Statistical comparison was performed using the Student's T-test in the statistical analysis software Prism (Graphpad, La Jolla CA).

Profiles of sarcomeric actin were produced as follows. IFM sarcomeres were stained with phalloidin as described above. Intensity profiles were measured from Z-disc to Z-disc using ImageJ (see Figure 10A,B). Overall sarcomere length was calculated as an average of Z-disc/Z-disc distance. Thin filament length was calculated as the distance from the Z-disc to halfway down the fluorescent intensity slope to the H-zone (Figure 10A). H-zone width was calculated as the distance between the end of one thin filament array to the beginning of the adjacent array (Figure 10A-B). Statistical analysis was performed using the Student's T-test in the statistical analysis software Prism (Graphpad, La Jolla CA).

### Quantitative PCR

Total RNA was isolated from >5 flies per experiment using TRIzol (Invitrogen). 1000 ng of total RNA was converted to cDNA using the qScript cDNA Synthesis Kit (Quanta BioSciences). qPCR was performed using the PerfeCta SYBR Green FastMix, ROX kit (Quanta Biosciences) with the primer pairs: 5′-TGAAGCCAGATTCAGCAAACCC-3′ and 5′-TACTGCCGGTAAACACCTCGTC-3′ for *bent*; 5′-ATGTCCGACGATGAAGAGTACACC-3′ and 5′-CTTCTGGTCCTGACGCTTGATG′3′ for *Mhc*; and 5′-AACTCGATCATGAAGTGCGACGTG-3′ and 5′-CTGCATACGATCGGCAATACCAG-3′ for *Act88F*. GAPDH mRNA levels were assayed as an internal control using the primer pair: 5′-AAAGCGGCAGTCGTAATAGC-3′ and 5′-GACATCGATGAAGGGATCGT-3′. Changes in expression were calculated using the comparative Ct method [82]. Both the wild-type profile and the RNAi-mediated knockdown experiments used at least 3 separate extractions 10 flies of total RNA at each time point. 1000 ng of total RNA was measured independently for each time point. Differences in mRNA levels were analysed using t-tests and the statistical analysis software Prism (Graphpad, La Jolla CA).

### Negative Geotaxis Assay

To quantify the climbing ability of wild-type flies (Figure 1E) and to identify climbing defects in our screen (Figures 3–5) [42] we used a negative geotaxis assay. The negative geotaxis assays were used to identify adult climbing defects in both the Mef2:GAL4 screen and the TARGET screen. Assays were carried out as previously described with modifications [27], [83]. Flies were collected immediately following eclosion and separated into batches of 10 in vials containing a standard food media. For the climbing assay, the flies from one vial were transferred to an empty vial with a line drawn 7.5 cm from the bottom. Flies were tapped to the bottom to induce an innate climbing response. The flies were scored as a ‘Success’ if they climbed above the 7.5 cm line or a ‘Failure’ if they did not. Five technical replicates were performed to ensure an accurate reading for each vial at each time-point. Approximately five biological replicate vials for each experimental line were tested at each time-point. Control flies with the appropriate expression system (Mef2:GAL4 or TARGET) but lacking an RNAi construct were assayed in parallel with the experimental line. Flies were assayed every three days from eclosion to 30 days post-eclosion. The initial time-point was assayed 24 hours post-eclosion to ensure the negative behavioural and neurological effects of carbon dioxide anaesthetization has subsided. Thus, the given time-points, reflect the number of days the RNAi construct had been expressed.

### Actin turnover analysis

A GFP-tagged Actin88F (Act88F::GFP) construct was obtained from the Bloomington Stock Center. Using the TARGET system, Act88F::GFP was induced post-eclosion in adult muscles. To track incorporation of Act88F::GFP into sarcomeres, a pulse-chase experiment was performed. Act88F::GFP was pulsed by moving flies to 29°C post-eclosion (Figure 2A). Optimization of the pulse-chase experiment determined that 28 hours was the ideal length for the pulse. Longer pulses of Act88F::GFP expression resulted in saturated level Act88F::GFP such that we could not observe a decline in fluorescent intensity. Shorter pulses did not allow fluorescent intensity reach the maximum as identified by continuous expression of Act88F::GFP. After the 28 hour pulse of Act88F::GFP, flies were returned to 18°C to limit Act88F::GFP expression. As a control, we also expressed a cytoplasmic UAS-eGFP (eGFP) under the same conditions. IFMs were dissected from fly thoraces and fixed ON at 4°C in 4% formaldehyde in 1x PBS.

To assess fluorescent intensity in the muscle for both eGFP and Act88F::GFP, we quantified sarcomeric fluorescent intensity in two ways. Firstly, we measured intensity at the Z-disc to determine the extent to which eGFP or Act88F::GFP was entering muscle fibres (Figure 2H, red boxes). Secondly, we calculated the ratio of the intensity at the Z-disc compared to the adjacent sarcomere body (Figure 2H, blue boxes), which included the entire sarcomere except the Z-disc. This allowed us to assess the primary location of incorporation. Measurements were obtained using ImageJ, and the data was processed using Prism (Graphpad, La Jolla CA).

### Bioinformatic analysis

The network analysis was performed using the software package Cytoscape (www.cytoscape.org) and the Drosophila Interaction Database (www.DroID.org). DroID provides a plug-in for Cytoscape that allows for the genetic and proteomic data for a set of genes to be mapped. Functional groupings were mined from the Gene Ontology.

Gene Ontology terms for the heatmap analysis were selected based on previous, similar studies [41]. Enrichment ratios were calculated by comparing frequency of a term in a specific phenotypic class to the frequency of the same term in the entire screened set. An enrichment ratio of 0 indicates that a given term did not appear in the phenotypic group. An enrichment ratio of <1 indicates that the frequency for the term was the reduced in the phenotypic class compared to the whole screened set. An enrichment ratio of 1 indicates that the frequency for the term was the same in the phenotypic class compared to the whole screened set. An enrichment ratio of >1 indicates that the frequency for the term was the enriched in the phenotypic class compared to the whole screened set.
